# Prevalence of abdominal aortic aneurysm (AAA) in first-degree relatives: detecting AAA in adult offspring of AAA patients

**DOI:** 10.1093/bjsopen/zrad163

**Published:** 2024-01-09

**Authors:** Nina Fattahi, Anneli Linné, Joy Roy, Malin Stenman, Sverker Svensjö, Olga Nilsson, Rebecka Hultgren

**Affiliations:** Department of Clinical Science and Education, Karolinska Institutet at Södersjukhuset, Stockholm, Sweden; Section of Vascular Surgery, Department of Surgery, Södersjukhuset, Stockholm, Sweden; Department of Clinical Science and Education, Karolinska Institutet at Södersjukhuset, Stockholm, Sweden; Section of Vascular Surgery, Department of Surgery, Södersjukhuset, Stockholm, Sweden; Department of Vascular Surgery, Karolinska University Hospital Stockholm, Stockholm, Sweden; Stockholm Aneurysm Research Group, STAR, Department of Molecular Medicine and Surgery, Karolinska Institutet, Stockholm, Sweden; Department of Molecular Medicine and Surgery, Karolinska Institutet, Stockholm, Sweden; Perioperative Medicine and Intensive Care Function, Karolinska University Hospital, Stockholm, Sweden; Department of Surgical Sciences, Uppsala University, Uppsala, Sweden; Department of Surgery, Falun County Hospital, Falun, Sweden; Department of Vascular Surgery, Karolinska University Hospital Stockholm, Stockholm, Sweden; Stockholm Aneurysm Research Group, STAR, Department of Molecular Medicine and Surgery, Karolinska Institutet, Stockholm, Sweden; Department of Vascular Surgery, Karolinska University Hospital Stockholm, Stockholm, Sweden; Stockholm Aneurysm Research Group, STAR, Department of Molecular Medicine and Surgery, Karolinska Institutet, Stockholm, Sweden

## Abstract

**Background:**

First-degree relatives of patients with abdominal aortic aneurysm (AAA) may have an increased risk of developing the disease. The primary aim was to report the prevalence of AAA in adult male and female offspring of patients with AAA. The secondary aim was to explore the efficiency of a registry-based detection route, and the third aim was to report contemporary prevalence in the population.

**Methods:**

Adult offspring of individuals with AAA and matched controls were identified through national registries. The examination included questionnaires and ultrasound examinations of the infrarenal aorta. Aortic pathology was defined as an aortic diameter ≥25 mm, AAA ≥30 mm.

**Results:**

The participation rate among male and female adult offspring was 64% (350/543) and 69% (402/583), respectively. A lower participation rate was found in male and female controls (51% and 52%). No difference in prevalence of AAA was observed between male adult offspring and controls (0.9%, c.i. 0.2 to 2.3%) or in the female population (prevalence of 0.2% in adult offspring and controls). Aortic pathology and previously diagnosed AAA were detected in 5.3% (c.i. 3.3 to 8.0%) of male adult offspring and 2.3% (c.i. 1.1 to 4.2%) in controls. Aortic pathology was more prevalent among adult offspring of females with AAA.

**Conclusion:**

The prevalence of AAA in the general population is low, but aortic pathology is notably higher among male first-degree relatives. Increased awareness should be directed towards individuals with a possible hereditary predisposition, particularly offspring of females with AAA and older smokers. Risk factor-based targeted screening of adult offspring of patients with AAA after registry-based detection should be further explored.

**Trial registration:**

ClinicalTrials.gov identifier, NCT4623268

## Teaser

First-degree relatives to aneurysm patients may have an increased risk of developing abdominal aortic aneurysm. Participants (1508 adult offspring with heredity for AAA and matched controls) were included through a register-based detection route. The results suggest that increased awareness should be directed towards individuals with a hereditary predisposition, particularly those with a female index person and older smokers. Risk factor-based targeted screening of adult offspring after registry-based detection should be further explored.

## Introduction

The benefits of discovering early-stage abdominal aortic aneurysm (AAA) were reported in RCTs of screening of elderly men in the 1990s^1,2^. These findings form the basis for the ongoing population-based screening programmes^3–6^. AAA is more common among men, who also develop the disease 5–10 years earlier than women; consequently, all cost-effectiveness analysis has failed to show benefits of population-based screening in women^7^. Hence, there is a lack of contemporary correct prevalence data for AAA in women^8^. With increasing attention drawn to influential gender differences in the care trajectory of AAA patients, an improved knowledge of standard aortic measurements can increase the quality of care^9,10^. In recent years, the 30 mm definition of AAA has been challenged, especially for women, and possible alternatives are consideration of the person’s body surface area and the aortic size (aortic size index, ASI), as well as aortic height index (AHI). Consideration of ASI and AHI in women compared with men could improve the precision in surveillance rather than only basing surveillance on maximum diameter. An expanded surveillance regime for sub-aneurysmal aortas (SAA; 25–29 mm) is reported in selected vascular clinics^10–12^.

The higher prevalence of AAA in first-degree relatives was first reported in 1977^13^, and later confirmed by others^2,14,15^. Family history is seen in approximately 15% (3–19%) of patients with AAA^16–20^. Prior reports also articulate a possible higher risk of developing AAA if the index person is a woman^16,21^. Environmental *versus* genetic components have been reported in twin registry studies, showing a heritability of 70% compared to environmental risks^22,23^. The European Society for Vascular Surgery recommends consideration of targeted screening for AAA in first-degree relatives of patients with AAA^2^.

The contemporary risk information regarding the risk of developing AAA among adult offspring is not based on scientific evidence, presumably due to practical methodological difficulties in tracking offspring at a relevant (older) age. There is consequently a need to explore new detection routes for patient groups with a late onset of hereditary diseases and, hitherto, national registries have not been evaluated for this.

The primary aim of this study was to investigate the prevalence of AAA in adult male and female offspring of patients with AAA compared to a control cohort without first-degree relatives with AAA or SAA. The secondary aim was to evaluate the feasibility of a registry-based detection route of adult offspring. The third aim was to report the contemporary prevalence of AAA and SAA in the male and female population.

## Methods

### Registry-based identification in national registries of the study population

The model of this cross-sectional study was reported in 2022^24^. The Swedish personal identity number^25^ was used by the National Board of Health and Welfare to retrieve information and assemble the study database. The selection was performed through the National Patient register, including the Swedish Inpatient and Outpatient registry data and the National Hospital Discharge registry, where individuals with AAA (born 1900–1953, index person) could be identified. In September 2020, 69 000 index persons were identified, the distribution of male and female ‘parents with disease’ was 75% and 25%, respectively. Index persons were linked to the Multigeneration register to identify offspring, 137 000 in total, of which 18 131 were registered in the County of Stockholm on 31 December 2019. Each offspring was matched by age, sex and region using Statistics Sweden to identify the control group. A random selection was performed in the identified offspring group of 18 131 individuals, with the mean age of 65 years for women and men, mirroring the age distribution in the population. This resulted in a cohort of 3800 individuals (2000 women and 1800 men adult offspring and controls). The first invitations were sent in October 2020, but halted from November 2020 to March 2021, followed by two other periods due to the COVID-19 pandemic. In June 2022 the study reached completion.

### Population-based screening in 65-year-old men

In Stockholm County, population-based screening of 65-year-old men was introduced in July 2010. By 2022, 130 000 men had been invited, of whom 110 000 had been examined and AAA had been detected in 1100 (unpublished data, personal communication with the screening programme in Stockholm, annual internal report).

### Study methodology and definitions

Invitations were sent out by randomly selecting individuals within the cohort of 3800. The invitation letter did not contain any information about the person’s specific first-degree relative^24^. Basic information was enclosed with a pre-booked ultrasound appointment, consent form, a personal code to access the web-based questionnaires and contact information. The included questionnaires were Hospital Anxiety and Depression Scale^26^, Euroqol-5D^27^ and some questions regarding their general health and awareness regarding AAA disease^24^. At the visit, the participants filled in the web-based forms, including questionnaires on demand supported by staff. The final step was the ultrasound examination mirroring the national screening programme for 65-year-old men, with the leading edge to leading edge ultrasound measurement method^5^. The ultrasound examination was conducted at the central core lab, performed with consecutive validation of the ultrasound quality. This core lab performs more than 8000 ultrasound examinations of the aorta annually in 65-year-old men. AAA is defined as aortic diameter (Ad) ≥30 mm, and SAA is defined as Ad 25–29 mm; together defined as aortic pathology.

The non-participant group consisted of five subgroups: non-responders after two invitations; declined participation after the first or second invitation; missing cases accounting for dead, emigrated, protected identity, unknown address, no longer resident in the county of Stockholm or dementia; non-participants who only received one invitation before the closure of the study; and excluded from the study, that is, those who had already undergone surgical intervention, already had known AAA, and in individuals where ultrasound examination could not be performed. As the assessment of the cost-effectiveness of a screening programme applies to detecting AAA/SAA within the programme, individuals already diagnosed with AAA were excluded from the main analysis. Persons who were invited and reported a diagnosed AAA were registered separately and are presented below.

### Sample size

The participation rate was expected to be clinically acceptable at approximately 65% (c.i. 63 to 67%), which is lower than in the population-based screening in men (75%) due to the lower age in invited adult offspring^11^. The large sex differences in AAA prevalence motivated the choice of a stratified analysis. For men with an alpha of 0.05, power 0.80, an estimated prevalence of 7.0% in offspring and 1.0% in controls, a required minimum of 166 persons in each group was estimated; considering 65% participation, 350 men were required in each group. For women with an alpha of 0.05, power 0.80, an estimated prevalence of 5.0% in offspring and 0.5% in controls, a required minimum of 206 persons in each group was requested and 400 women in each group was decided.

### Outcome

The primary outcome was the prevalence of AAA and SAA in adult female and male offspring of individuals with AAA and in a matched control group. The secondary outcome was the participation rate in adult offspring and controls, for women and men.

### Statistical analysis

Self-reported risk factors and baseline characteristics were described with descriptive statistics, for women and men separately. Continuous variables were reported as means and standard deviations and *t*-tests were used to test for differences. Categorical data were reported in proportions and statistical significance was tested with a chi-squared test or Fisher’s test where appropriate. The analysis was mainly descriptive, but with some comparisons: female adult offspring and matched controls; male adult offspring and matched controls; participants with Ad 25–29 mm and ≥30 mm; subgroup analysis per age bracket (65–80, 65–75, 65–70 years); adult offspring and controls; and adult offspring and controls regarding Ad, ASI, and AHI both including and excluding individuals with AAA. ASI was calculated as Ad (cm) divided by body surface area (BSA) (m^2^)^28^, whereas BSA was calculated according to the Dubois and Dubois formula^29^. AHI is calculated by the Ad (cm) divided by height in metres^10,30^.

### Reporting and ethical approval

The study was approved by the Swedish Ethical Review Authority (Dnr: 2019-01076) and registered at the website of Clinical Trials (ClinicalTrials.gov:identifier, NCT4623268). The results are reported according to the STROBE reporting guidelines^31^.

## Results

### Participation rate

**Fig. 1:**
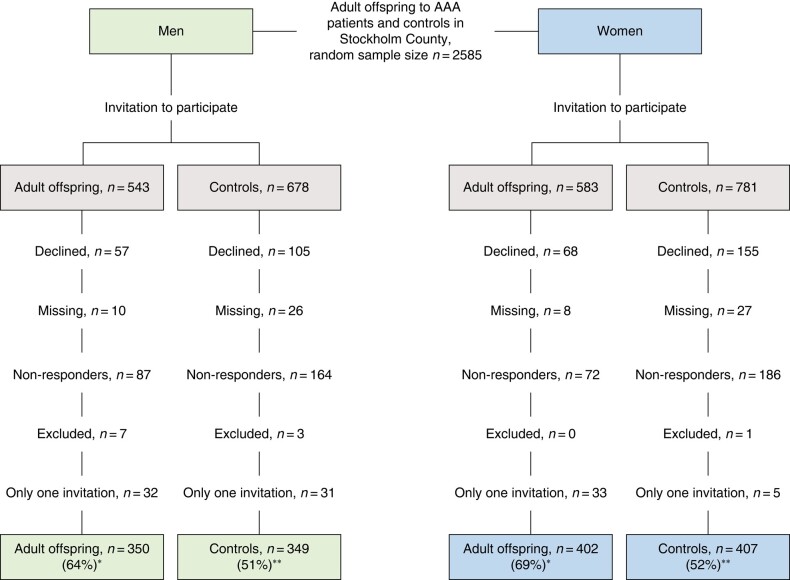
Overview of enrolment process and participation rate for men and women *The ratio is calculated as adult offspring participation/adult offspring invited. **The ratio is calculated as controls participation/controls invited.

Overall, 1508 participants were included (699 men, 809 women), with an age range of 45–80 years for men and 50–80 years for women. The participation rate was higher for the adult offspring than for the controls, both in men (64% *versus* 51%) and women (69% *versus* 52%) (*Fig. 1*). The group of non-participants was dominated by the control group, non-responders and persons declining participation, and their distribution is shown in *Table S1*.

### Study cohort characteristics

Most characteristics were similar when comparing the male adult offspring and the control group (*n* = 699), and this pattern was mirrored in women (*n* = 809) (*Tables 1*, *2*). The distribution of risk factors was similar for both women and men. Both in the male and female adult offspring group a higher mean height was reported compared to controls (*P* = 0.019 and *P* = 0.012, respectively), and a higher mean aortic size was detected in male offspring (*P* = 0.011) (*Tables 1*, *2*; *Fig. S1*). The participants’ characteristics were also analysed with no consideration of sex. The adult offspring had a higher prevalence of prior venous thromboembolism and controls had a higher prevalence of prior myocardial infarction. Height, Ad and AHI had a higher mean among adult offspring than controls (*Table S2*; *Fig. S1*).

**Table 1:** Characteristics of male adult offspring of abdominal aortic aneurysm patients and controls including aortic measurements

Men	Adult offspring (*n* = 350)	Controls (*n* = 349)	*P*
Age (years), mean(s.d.)	63.3(7.9)	64.3(8.3)	0.121
Height (cm), mean(s.d.)	180.7(7.4)	179.2(9.4)	0.019
Weight (kg), mean(s.d.)	88.7(14.9)	86.5(12.9)	0.038
BMS (kg/m^2^), mean(s.d.)	27.1(4.0)	27.2(7.2)	0.883
Abdominal aortic diameter (mm), mean(s.d.)	18.9(4.1)	18.1(3.5)	0.011
Aortic size index (cm/m^2^), mean(s.d.)	0.91(0.21)	0.89(0.20)	0.186
Aortic height index (cm/m), mean(s.d.)	1.04(0.24)	1.01(0.21)	0.040
**Smoking status**			0.844
Never	169 (48)	166 (48)	
Former	156 (45)	162 (46)	
Current	24 (7)	21 (6)	
Hypertension	165 (47)	144 (42)	0.117
Diabetes mellitus	47 (14)	40 (12)	0.422
Angina pectoris	10 (3)	18 (5)	0.117
Heart failure	13 (4)	23 (7)	0.085
Kidney failure	7 (2)	8 (2)	0.794
Pulmonary disease	22 (6)	17 (5)	0.415
Bleeding disorder	1 (0.3)	0 (0.0)	0.317
Prior venous thrombosis	24 (6.9)	23 (6.6)	0.880
Prior myocardial infarction	11 (3)	21 (6)	0.070
Prior stroke	15 (4)	17 (5)	0.730
Antihypertensive treatment	155 (45)	150 (43)	0.678
Lipid-lowering therapy	103 (30)	83 (24)	0.075
Anti-platelet therapy/anticoagulant medication	63 (18)	73 (21)	0.349
Other medications	148 (44)	149 (46)	0.719

Values are *n* (%) unless otherwise stated. Information regarding smoking and co-morbidity was collected by self-reported questionnaires prior to the ultrasound examination.

**Table 2:** Characteristics of female adult offspring of abdominal aortic aneurysm patients and controls including aortic measurements

Women	Adult offspring (*n* = 402)	Controls (*n* = 407)	*P*
Age (years), mean(s.d.)	64.8(7.5)	64.4(7.6)	0.535
Height (cm), mean(s.d.)	166.7(6.1)	165.5(6.6)	0.012
Weight (kg), mean(s.d.)	71.4(14.4)	70.4(12.7)	0.319
BMS (kg/m^2^), mean(s.d.)	25.7(4.9)	25.7(4.5)	0.920
Abdominal aortic diameter (mm), mean(s.d.)	16.5(2.2)	16.3(2.1)	0.251
Aortic size index (cm/m^2^), mean(s.d.)	0.93(0.14)	0.92(0.14)	0.702
Aortic height index (cm/m), mean(s.d.)	0.99(0.15)	0.99(0.12)	0.228
**Smoking status**			0.186
Never	183 (46)	198 (49)	
Former	179 (45)	184 (45)	
Current	37 (9)	24 (6)	
Hypertension	145 (36)	148 (36)	0.952
Diabetes mellitus	23 (6)	31 (8)	0.284
Angina pectoris	11 (3)	16 (4)	0.347
Heart failure	9 (2)	10 (3)	0.838
Kidney failure	4 (1)	4 (1)	0.989
Pulmonary disease	33 (8)	36 (9)	0.738
Bleeding disorder	5 (1)	3 (1)	0.464
Prior venous thrombosis	29 (7)	12 (3)	0.006
Prior myocardial infarction	5 (1)	12 (3)	0091
Prior stroke	10 (2)	13 (3)	0.545
Antihypertensive treatment	135 (34)	146 (36)	0.494
Lipid-lowering therapy	70 (18)	68 (17)	0.789
Anti-platelet therapy/anticoagulant medication	43 (11)	53 (13)	0.294
Other medications	212 (54)	212 (54)	0.846

Values are *n* (%) unless otherwise stated. Information regarding smoking and comorbidity was collected by self-reported questionnaires prior to the ultrasound examination.

### Prevalence of abdominal aortic aneurysm

The prevalence of AAA (≥30 mm) was 0.9% among male adult offspring and controls (3/350 *versus* 3/349; *Table 3*). There were nine participants with SAA in male adult offspring and two in controls with a RR estimate of 4.5 (c.i. 1.0 to 20.6) for adult offspring (*Table 3*). The prevalence of AAA and SAA was 12/350 (3.4%) in male adult offspring and 5/349 (1.4%) in controls, the RR was 2.4 (c.i. 0.9 to 6.7) for adult offspring to be diagnosed with any infrarenal aortic pathology (*Table 3*).

**Table 3:** Identified abdominal aortic aneurysm (AAA) and sub-aneurysmal aorta (SAA) among adult offspring of AAA patients and the control group

		95% c.i. for proportions	Relative risk	ASI (cm/m^2^), mean	AHI (cm/m), mean
**AAA men**	**≥30 mm, *n* (%)**				
Adult offspring, *n* = 350	3 (0.9)	(0.2%,2.3%)		2.5	3.2
Control, *n* = 349	3 (0.9)	(0.2%,2.3%)		2.4	2.6
**SAA men**	**25–29 mm, *n* (%)**				
Adult offspring *n* = 350	9 (2.6)	(1.3%,4.6%)	4.5 (c.i. 1.0,20.6)	1.3	1.5
Control, *n* = 349	2 (0.6)	(0.1%,1.8%)		1.2	1.4
**AAA + SAA men**	**≥25 mm, *n* (%)**				
Adult offspring, *n* = 350	12 (3.4)	(1.9%,5.7%)	2.4 (c.i. 0.9,6.7)	1.6	2.1
Control, *n* = 349	5 (1.4)	(0.5%,3.1%)		1.9	2.0
**AAA women**	**≥30 mm, *n* (%)**				
Adult offspring, *n* = 402	1 (0.2)	(0%,1.2%)		2.0	2.1
Control, *n* = 407	1 (0.2)	(0%,1.1%)		1.8	1.9
**SAA women**	**25–29 mm, *n* (%)**				
Adult offspring, *n* = 402	2 (0.5)	(0.1%,1.6%)	2 (c.i. 0.2,22.2)	1.8	1.7
Control, *n* = 407	1 (0.2)	(0%,1.1%)		1.7	1.7
**AAA + SAA women**	**≥25 mm, *n* (%)**				
Adult offspring, *n* = 402	3 (0.7)	(0.2%,2.0%)	1.5 (c.i. 0.3,9)	1.8	1.6
Control, *n* = 407	2 (0.5)	(0.1%,1.6%)		1.8	1.6

ASI, aortic size index; AHI, aortic height index.

The prevalence of AAA among female adult offspring and controls was similar (*Table 3*). In female adult offspring, two were identified with SAA (25 and 29 mm, respectively), compared to one control (2/402 *versus* 1/407) (*Table 3*). The prevalence of AAA and SAA was 3/402 (0.7%) in female adult offspring and 2/402 (0.5%) in controls with an RR of 1.5 (c.i. 0.3 to 9) (*Table 3*).

### Age and smoking

The mean age of participants diagnosed with AAA among the male adult offspring was 73.3 years and in controls 71.3 years (*Table 4*). More men were diagnosed among the adult offspring in the age-stratified analysis of 65–80 years old (2% *versus* 1.2% in controls); these men constituted 46% of the whole male cohort. Including all AAA and SAA at 65–80 years, the prevalence among adult offspring was higher (7.4%, 11/149 *versus* 2.3%, 4/171). The RR to develop any aortic pathology for male offspring was 3.2 (c.i. 1.0 to 9.7) (*Table 4*).

**Table 4:** Identified abdominal aortic aneurysm (AAA) and sub-aneurysmal aorta (SAA) among male and female adult offspring to AAA patients and controls presented for different age groups

		AAA, *n* (%)	95% c.i. for proportions	AAA + SAA, *n* (%)	95% c.i. for proportions	AAA + SAA, RR (c.i.)	AD (mm), mean(s.d.)	Age (years), mean(s.d.)
**Men age interval**								
65–80	Adult offspring (*n* = 149)	3 (2%)	(0.6%,5.3%)	11 (7.4%)	(4%,12.4%)	3.2 (c.i. 1.0,9.7)	20(6)	71.0(4.3)
	Control (*n* = 171)	2 (1.2%)	(0.2%,3.7%)	4 (2.3%)	(0.8%,5.5%)		19(5)	71.0(4.4)
65–75	Adult offspring (*n* = 119)	2 (1.7%)	(0.4%,5.3%)	7 (5.9%)	(2.7%,11.2%)	3.8 (c.i. 0.8,17.8)	19(5)	69.5(3.1)
	Control (*n* = 128)	0		2 (1.6%)	(0.3%,4.9%)		18(2)	69.4(3.0)
65–70	Adult offspring (*n* = 60)	0		1 (1.4%)	(0.1%,6.2%)		19(3)	67.4(1.9)
	Control (*n* = 71)	0		0			18(2)	67.5(1.7)
45–65	Adult offspring (*n* = 201)	0		1 (0.5%)	(0.1%,2.3%)		18(2)	58.0(4.2)
	Control (*n* = 178)	1 (0.6%)	(0.1%,2.6%)	0			18(2)	57.0(4.4)
**Women age interval**								
65–80	Adult offspring (*n* = 187)	1 (0.5%)	(0.1%,2.5%)	3 (1.6%)	(0.5%,4.2%)	1.5 (c.i. 0.3,8.8)	17(3)	71.6(4.2)
	Control (*n* = 186)	1 (0.5%)	(0.1%,2.5%)	2 (1.1%)	(0.2%,3.4%)		16(2)	71.5(4.4)
65–75	Adult offspring (*n* = 151)	1 (0.7%)	(0.1%,3.1%)	3 (2.0%)	(0.6%,5.2%)	1.4 (c.i. 0.2,8.4)	17(3)	70.1(3.1)
	Control (*n* = 144)	1 (0.7%)	(0.1%–,3.2%)	2 (1.4%)	(0.3%,4.4%)		17(3)	69.6(3.1)
65–70	Adult offspring (*n* = 79)	0		0			16(2)	67.6(1.8)
	Control (*n* = 83)	0		1 (1.2%)	(0.1%,5.5%)		16(3)	67.3(1.8)
50–65	Adult offspring (*n* = 215)	0		0			16(2)	59.0(4)
	Control (*n* = 221)	0		0			16(2)	58.0(4)

RR, relative risk; AD, aortic diameter.

In the female population the mean age among individuals with AAA was 74 years for adult offspring and 71 years for controls (*Table 4*). Including all AAA and SAA for women at 65–80 years, the prevalence among adult offspring was 1.6% *versus* 1.1% in controls. The RR was 1.5 (c.i. 0.3 to 8.8) in females with heredity (*Table 4*).

**Fig. 2:**
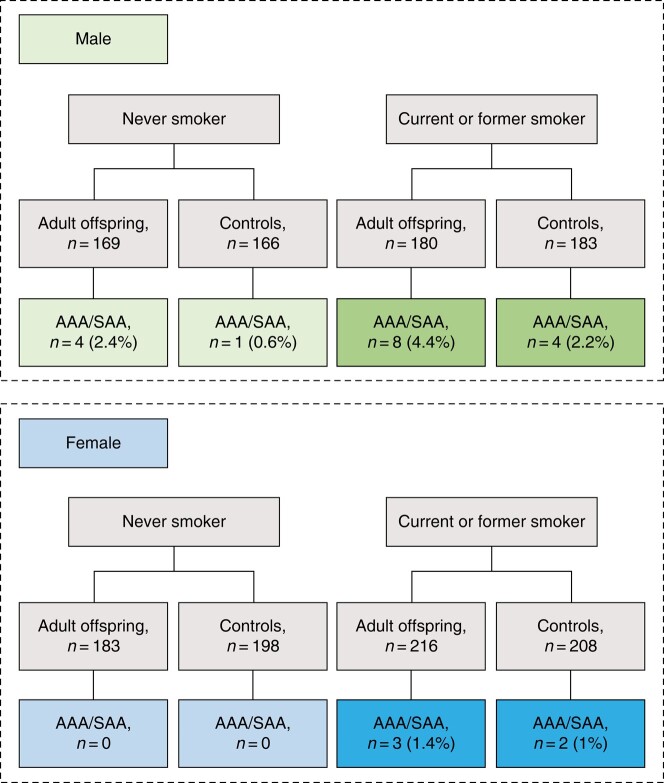
Distribution of abdominal aortic aneurysm (AAA) and sub-aneurysmal aorta (SAA) among male/female adult offspring to individuals with AAA and controls Never (*n* = 5)/ever smoker (*n* = 17).

The patients diagnosed with AAA among the male offspring were all former smokers; among the controls there were one current, one former and one non-smoker (*Fig. 2*). Regarding the nine SAA in the male adult offspring, there were four non-smokers and five former smokers. The two SAA among controls were former smokers. All identified AAA and SAA female persons were current or former smokers (*Fig. 2*).

### Index person: mother or father with abdominal aortic aneurysm

**Fig. 3:**
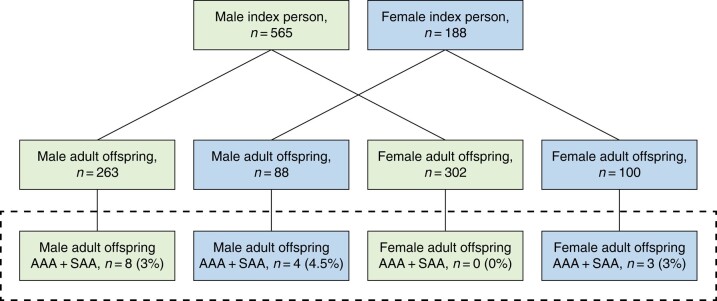
Distribution of abdominal aortic aneurysm (AAA) and sub-aneurysmal aorta (SAA) disease sorted by the sex of the index person (the parent) presented for adult offspring women and men

The association between the sex of the index person (the mother or father) and the occurrence of AAA or SAA in the adult offspring is shown in *Fig. 3*. A higher proportion was detected in offspring of a female index person (3/100 daughters and 4/88 sons to mothers with AAA) compared to male index persons (0/302 daughters and 8/263 sons to fathers with AAA; *Fig. 3*).

### Aortic size index and aortic height index

The mean AHI differed between the adult offspring and controls (*Table S2*). In the male population the AHI was higher in the adult offspring (1.04 (s.d. 0.24)) compared to the control group (1.01 (s.d. 0.21)) (*P* = 0.040), but no difference was noted in the females (*Tables 1, 2*). In the male population the SAA had a mean ASI of 1.30 in the adult offspring and 1.20 in the control group. In the female adult offspring, the identified SAA had a mean ASI of 1.78, and in the female control group one SAA was identified with an ASI of 1.72 (*Table 3*). The ASI among the male and female adult offspring for the overall group, as well as for the examined group only including Ad < 30 mm, did not differ from the controls. The AHI remained higher in the adult offspring group, both when including AAA individuals as well as excluding them (*Table S3*).

### Prior diagnosis

In male adult offspring, seven individuals reported that they had been diagnosed with AAA (mean age 73 years). Six of them were already treated and one diagnosed; these were not included in the study. Among the male controls, three individuals reported that they had been treated (mean age 74 years). Among the 10 individuals diagnosed prior to invitation, four had been diagnosed in the screening programme for 65-year-old men.

Re-evaluating the AAA prevalence by including the above-mentioned seven adult male offspring and three controls, the prevalence in male adult offspring was 2.8% (10/357) and in controls 1.7% (6/352). The prevalence of any aortic pathology at any time period was higher in the male adult offspring (19/357 (5.3%) *versus* 8/352 (2.3%)). The RR for adult offspring to have any pathology was a 2.3 higher risk of developing AAA in comparison to controls. No women reported prior diagnosis of AAA.

## Discussion

This exploratory cross-sectional study provides the contemporary prevalence of AAA and SAA in male and female adult offspring of patients diagnosed with AAA, as well as for men and women in the general population. Detailed analysis supports prior knowledge that individuals older than 65 years, males and smokers have an increased risk of developing AAA compared to the general population. This is more evident in adult offspring of patients with AAA. Once again, the considerable sex difference in the disease distribution is confirmed. The results also validate the feasibility to obtain adequate participation rates in a targeted screening model when using national registries as detection routes.

In the regional screening programme, a declining rate of detected AAA from the start in 2010 until 2022 has been observed, from a prevalence of 1.4% in 2010 to 0.8% (unpublished data, personal communication), with a non-declining participation rate of 75%. The low prevalence of AAA in the offspring group, in comparison to prior publications^16–18,32^, is somewhat surprising. Several possible explanations could contribute: the decline in smoking on a national level and better adherence to secondary prevention programmes for cardiovascular risk groups, as shown by the declining mortality of coronary heart disease by 56% between 1986 and 2002^33^, as well as the lower mean age in participants. The latter is partly confirmed: when excluding participants younger than 65 years, a higher prevalence was noted (2.0% *versus* 1.2%). When extending the analysis to include individuals with already known AAA, the prevalence increases further, which is more in line with earlier reports on the increased risk of the hereditary component, reaching 5.3% (2.3% controls). Hence, this could be considered as the true prevalence in this risk group. It is possible that persons with awareness of their hereditary risk could have been selectively screened at a younger age^34^.

SAA has been reported to be a possible group to include in surveillance due to the high risk of later developing an AAA^12,35^. The prevalence increases considerably among the male adult offspring when this aortic pathology is included, specifically individuals above 65 years. What appears evident is that individuals with AAA or SAA share the same cardiovascular risk burden and targeted secondary prevention could be an important strategy for persons with smaller AAA and SAA. More longitudinal studies would be required to support the cost-effectiveness for such targeted screening with wider diagnostic criteria.

The mean AHI and Ad are higher in male adult offspring, which may indicate that these individuals have a generally larger aorta (illustrated in *Fig. S1*). There is a lack of similar findings in the female population, which also could be due to the low proportion of aortic pathology overall in women. Women are not considered for inclusion in population-based screening^7^. This has been challenged by reports on high prevalence in specific risk groups^8,17^. The prevalence seen in the current study does not support a re-evaluation of present screening guidelines in women, when an Ad ≥30 mm is used.

The use of alternative methods, such as ASI, has also been suggested, and a higher prevalence was detected in the female adult offspring compared to controls when this was applied (0.7% *versus* 0.5%). There is a lack of contemporary evidence to support this measurement as a basis for population-based screening, but future analysis of cost-effectiveness should include these measurements. These data clearly show that the sex differences are less profound when applying ASI or AHI as compared to diameter (*Fig. S1*). There is a standard limit (ASI≥1.5) that can be used as a threshold indicating very small aneurysms for prolonged surveillance^36^.

It has previously been reported that there is an increased risk for AAA if the index person (the parent) is female^21,32^. This report provides some support to such a theory, and targeted familial screening of first-degree relatives of women who present with AAA could be clinically relevant.

Due to the methodological challenges of detecting adult offspring, at a higher age, few investigations have been performed historically. By exploring the feasibility of merging several national registries and performing an ultrasound examination in targeted participants, this ‘Detecting AAA in adult offspring of AAA patients’ (DAAAD) model has been proven to work. The participation rate is lower than the contemporary national screening programmes^37–39^, possibly because the study was performed amid the COVID-19 pandemic. The higher attendance rates in these programmes are presented for periods before the pandemic and are still not available for this time period; thus, the attendance rate is expected to mirror that of the other programmes during the pandemic. A difference in the participation rates between adult offspring and controls was expected, even though the invitation sent was neutral regarding any hereditary risk. It has previously been reported that the prevalence of AAA is commonly higher in non-participating population groups. However, the higher participation rate among the offspring of patients with AAA compared to controls suggests that awareness of the risk could increase the willingness to be screened in this population. Altogether, there can be some underestimation of the overall prevalence.

One factor that has been reported to influence the hereditary risk is the risk of shared familial risk factors, such as smoking. The examined participants had a very similar risk factor profile. This group represents an average cohort of Swedish persons, in line with the recently published data from the SCAPIS cohort, a population-based contemporary screening of 35 000 persons in the population^40^.

The age interval in the study design may introduce some limitation regarding the clinical interpretation of the results. The inclusion of men and women as young as 45 and 50 years, respectively, is due to the fact that hereditary AAA may present differently in terms of disease progression and earlier onset as for other hereditary diseases and this might be a contributing factor for the low prevalence overall^34^. The strong overall risk in 65-year-old men with SAA to proceed to AAA and above-threshold for repair within a 5–10-year period is important to include when examining the prevalence of aortic pathology, especially in a cohort including younger persons in a ‘screening’ project^35,41^. It would be deemed unethical in a population-based screening study to perform ultrasound examination in younger persons and disregard the 25–29 mm SAA with this ultrasound without considering their life-long risk. This is especially true for women of course.

Given that the index persons were born between 1900 and 1953, there could be a possibility that a few index persons in the control group have undiagnosed AAA, especially the men born 1900–1942. Screening was introduced in 2006 for 65-year-old men. The prevalence of AAA was approximately 1.5% in 2010 in 65-year-old men, which could mean that 3–5 persons had undetected AAA in the control group considering the low mean age.

The results of this study are likely to be applicable on a nationwide level. The prevalence of AAA has some national variations and minor regional differences are noticeable, also within population-dense regions like Stockholm. The reported differences in prevalence between the sexes, the association between high prevalence with increasing age and among smokers, confirms the validity of these results. Even if the results presumably are generalizable regarding the distribution between the sexes, or the adult offspring and controls, the findings would differ depending on the underlying prevalence of AAA in the population as such, but probably the ratio would be quite similar. It is of course more challenging to transfer the study design based on national registries to countries lacking a multigeneration register, but it is possible to identify family trees by other methods, such as hospital registries, other governmental registries or insurance registries.

Overall, the prevalence of AAA in men and women in the general population is low, but the prevalence of aortic pathology, including previously diagnosed AAA, in persons with possible hereditary risks is notably higher. This result is further amplified when only considering the population over 65 years. These findings might be important to consider in the context of the observed declining prevalence rates in the male 65-year-old population, regarding long-term cost-effectiveness of the population-based screening programmes. The feasibility of this registry-based detection model proved to be successful with an adequate attendance rate, and this study contributes a new design model for detecting risk groups with hereditary diseases for future targeted screening programmes. As well as reporting AAA prevalence in first-degree relatives of individuals with AAA, a contemporary prevalence of AAA and SAA in a female and male population is reported, including newer models of reporting aortic size, such as ASI and AHI.

## Supplementary Material

zrad163_Supplementary_Data

## Data Availability

There is no general access to data files.
