# Supplementary material for: Prevalence of abdominal aortic aneurysm (AAA) in first-degree relatives: detecting AAA in adult offspring of AAA patients
Source: BJS Open. 2024 Jan 9;8(1):zrad163. doi: 10.1093/bjsopen/zrad163 (PMC10776345; doi:10.1093/bjsopen/zrad163)
Supplement: zrad163_Supplementary_Data [file zrad163_supplementary_data.docx]

**Title: Prevalence of Abdominal Aortic Aneurysms (AAA) in first degree relatives**

**- Detecting AAA in adult offspring of AAA patients**

Authors: Nina Fattahi^1,2^, Anneli Linné^1,2^, Joy Roy^3,4^, Malin Stenman^5,6^, Sverker Svensjö^7,8^, Olga Nilsson^3,4^, Rebecka Hultgren^3,4^

1. Dept of Clinical Science and Education, Karolinska Institutet at Södersjukhuset, Stockholm, Sweden.
2. Section of Vascular Surgery, Department of Surgery, Södersjukhuset, Stockholm, Sweden.
3. Department of Vascular Surgery, Karolinska University Hospital Stockholm, Sweden.
4. Stockholm Aneurysm Research group, STAR, Department of Molecular Medicine and Surgery, Karolinska Institutet, Stockholm, Sweden.
5. Department of Molecular Medicine and Surgery, Karolinska Institutet, Stockholm, Sweden.
6. Perioperative Medicine and Intensive Care Function, Karolinska University Hospital, Stockholm, Sweden.
7. Department of Surgical Sciences, Uppsala University, Uppsala, Sweden
8. Centre for Clinical Research, Falun, Sweden

**Corresponding author:** Corresponding author Rebecka Hultgren

[Rebecka.hultgren@ki.se](mailto:Rebecka.hultgren@ki.se)

Address: Karolinska Institutet, Department of Vascular Surgery, S3:01 Karolinska University Hospital, 171 76 Stockholm, Sweden

ORCID ID; 0000-0002-8869-0493

**Supplementary Materials - Index**

| **Supplementary Methods** |  |
| --- | --- |
| Not applicable |  |
| **Supplementary Results** |  |
| Not applicable |  |
| **Supplementary Appendixes** |  |
| Not applicable |  |
| **Supplementary Figures and Tables** |  |
| Table S1 | *Pag.3* |
| Table S2 | *Pag.4* |
| Table S3  Supplemental figure 1 | *Pag.5*  *Pag.6* |
| Graphical Abstract | *Pag.7* |
| **References** |  |
| Not applicable |  |

**Supplementary Figures and Tables**

**Supplemental Tables**

**Table S1**. The distribution of non-participants in the study between men/women and adult offspring/control.

| **Non-participants** | **Non-responders** | **Participation declined** | **Missing** | **Only one invitation** | **Excluded** | **Total non-participants** | **Mean age** |
| --- | --- | --- | --- | --- | --- | --- | --- |
| **Men** |  |  |  |  |  |  |  |
| Adult offspring | 87 | 57 | 10 | 32 | 7 | **193** | 64.7 |
| Control | 164 | 105 | 26 | 31 | 3 | **329** | 62.8 |
| **Total** | **251** | **162** | **36** | **63** | **10** | **522** |  |
| **Women** |  |  |  |  |  |  |  |
| Adult offspring | 72 | 68 | 8 | 33 | 0 | **181** | 65.6 |
| Control | 186 | 155 | 27 | 5 | 1 | **374** | 64.3 |
| **Total** | **258** | **223** | **35** | **38** | **1** | **555** |  |

**Table S2.** Characteristics of the 1508 participants in the study, including aortic size diameter. Information regarding smoking and comorbidity is collected by self-reported questionnaires prior to ultrasound examination.

| **Women and men** | **Adult offspring (n=752)** | **Controls**  **(n=756)** | **P-value** |
| --- | --- | --- | --- |
| Age, mean years (SD) | 64.1(7.7) | 64.4 (8.0) | 0.551 |
| Height, mean cm (SD) | 173.1 (10.5) | 172 (9.8) | 0.034 |
| Weight, mean kg (SD) | 79.4(17) | 77.8 (15.1) | 0.059 |
| BMI, mean kg/m2 (SD) | 26.6 (7.8) | 26.2 (4.2) | 0.216 |
| Abdominal aortic diameter, mean mm (SD) | 17.6 (3.4) | 17.1 (3.0) | 0.008 |
| Aortic size index, mean cm/m2 (SD) | 0.92 (0.18) | 0.91 (0.16) | 0.154 |
| Aortic height index, mean cm/m (SD) | 1.02 (0.20) | 1.0 (0.17) | 0.007 |
| Smoking status |  |  | 0.251 |
| - Never | 61(8%) | 45 (6%) |  |
| - Former | 352 (45%) | 364 (46%) |  |
| - Current | 335 (47%) | 346 (48%) |  |
| Hypertension | 310 (41.4%) | 292 (38.7%) | 0.292 |
| Diabetes mellitus | 70 (9.3%) | 71 (9.4%) | 0.969 |
| Angina pectoris | 21(2.8%) | 34 (4.5%) | 0.077 |
| Heart failure | 22 (2.9%) | 33 (4.4%) | 0.136 |
| Kidney failure | 11 (1.5%) | 12 (1.6%) | 0.844 |
| Pulmonary disease | 55 (7.3%) | 53 (7.0%) | 0.825 |
| Bleeding disorder | 6 (0.8%) | 3 (0.4%) | 0.310 |
| Prior venous thrombosis | 53 (7.1%) | 35 (4.6%) | 0.044 |
| Prior myocardial infarction | 16 (2.1%) | 33 (4.4%) | 0.014 |
| Prior stroke | 25 (3.3%) | 30 (4.0%) | 0.515 |
| Antihypertensive treatment | 290 (38.8%) | 296 (39.3%) | 0.847 |
| Lipid lowering therapy | 173 (23.3%) | 151 (20.1%) | 0.136 |
| Anti-platelet therapy/anticoagulant medication | 106 (14%) | 126 (17%) | 0.166 |
| Other medications | 360 (49.8%) | 361 (50.1%) | 0.895 |
|  |  |  |  |

*BMI (Body mass index); categorical variables are presented as number (%). Continuous variables are presented as mean (standard deviation).*

**Table S3.** Maximum Aortic diameter (AD) and corresponding Aortic Size Index (ASI) and Aortic Height Index (AHI) in the whole cohort (n=1508), presented as mean (standard deviation)

| **Men and women n=1508** | **Adult offspring (n=752)** | **Controls (n=756)** | **P-value** |
| --- | --- | --- | --- |
| **Age, mean years (SD)** | 64.1 (7.7) | 64.4 (8.0) | 0.551 |
| **AD, mean mm (SD)** | 17.6 (3.4) | 17.1 (3.0) | 0.008 |
| **ASI, mean cm/m2) (SD)** | 0.92 (0.18) | 0.91 (0.16) | 0.154 |
| **AHI, mean cm/m (SD)** | 1.02 (0.20) | 1.0 (0.17) | 0.034 |
| **ASI (AAA excluded), mean cm/m2 (SD)** | 0.92 (0.13) | 0.90 (0.12) | 0.086 |
| **AHI (AAA excluded), mean cm/m (SD)** | 1.0 (0.14) | 0.99 (0.11) | 0.007 |

**Supplemental Figure 1.** The aortic diameter, aortic size index and aortic height index are presented for all investigated individuals (adult offspring and controls). The results are also presented for women (blue) and men (orange) separately. The figure includes all measurements below a maximum diameter of 3 cm.

*
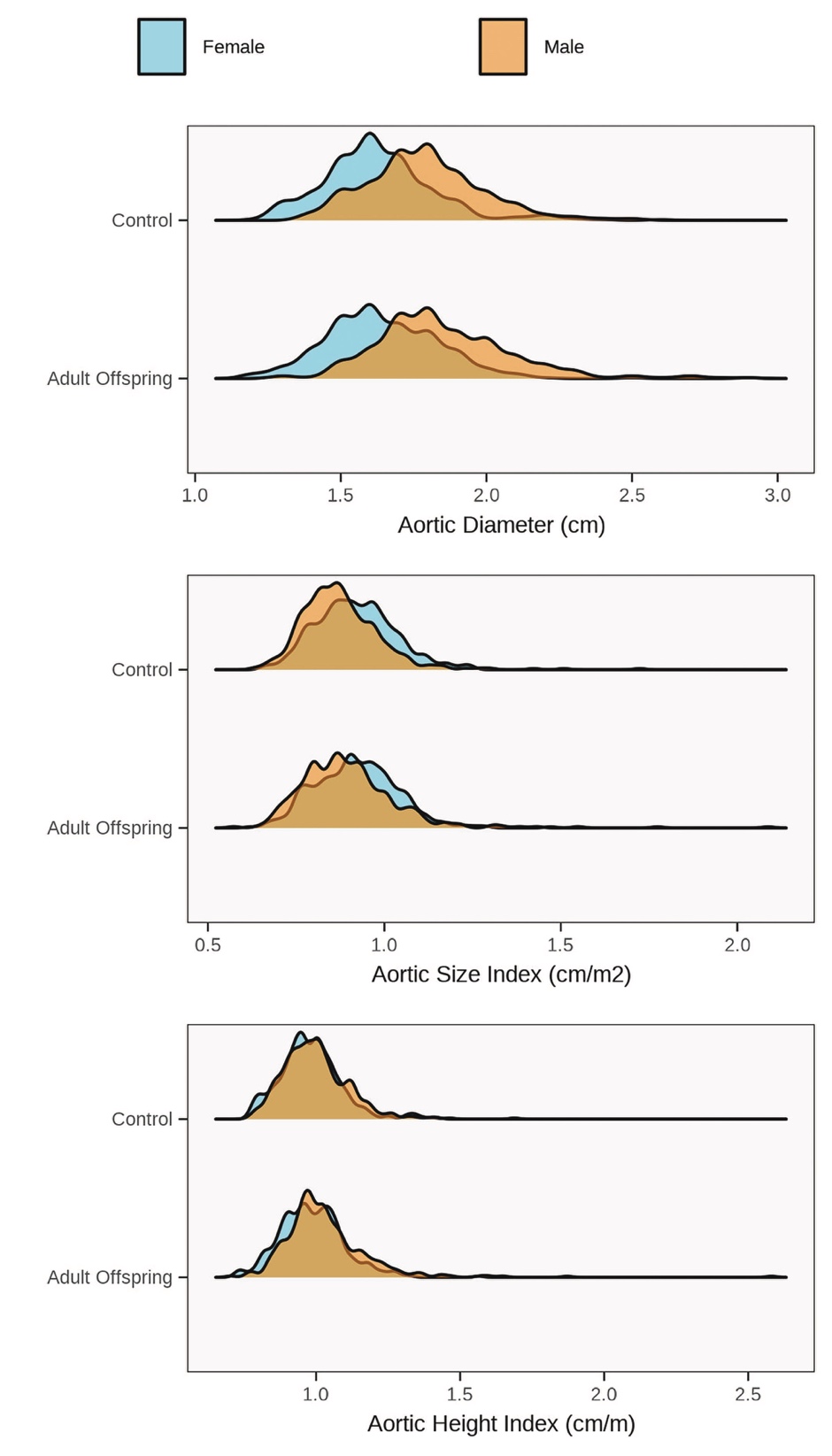
*

**Graphical Abstract.** Prevalence of AAA and SAA among male and female adult offspring compared to controls. Participation rate among male and female adult offspring and controls. AAA (abdominal aortic aneurysm), SAA (sub-aneurysmal aorta)

**
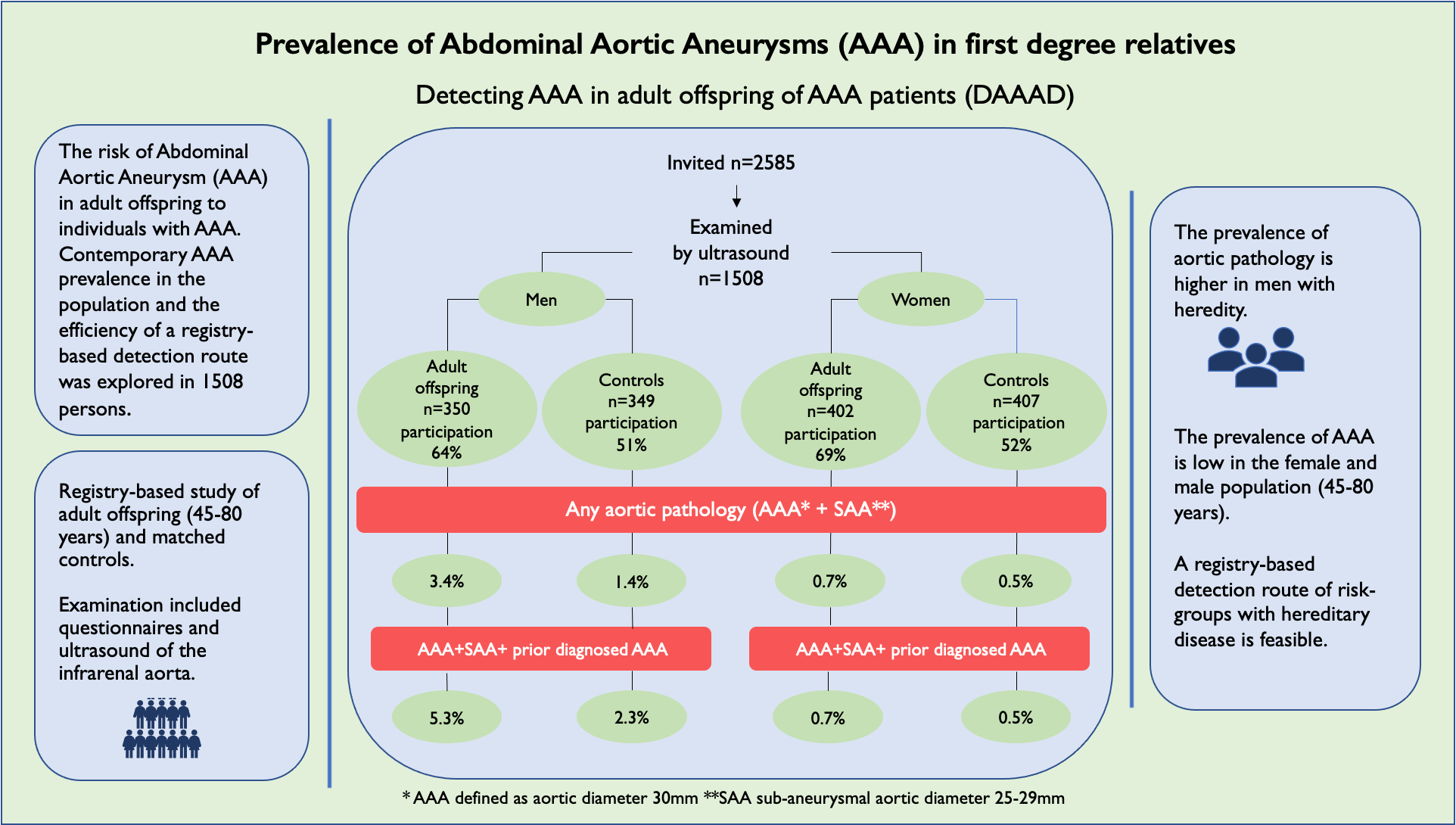
**
